# Notes and News

**Published:** 1894-12-15

**Authors:** 


### NOTES AND NEWS.

COLLECTED AND COMMUNICATED.

A new hospital, the Hospital of St. Joseph, has just been opened at Lyons. It is to accommodate 120 patients and to be entirely under Roman Catholic management, and will, it is expected, result in the establishment of a Catholic Faculty at Lyons.

Writing of the Japanese Hospital, near Nagasaki, a correspondent to the press expresses great admiration. He says it was equipped with the best modern instruments and newest antiseptics, whilst the whole hospital was conducted on an excellent and modern system. The medical staff were all Japanese, who had graduated in medicine and surgery either in America or England, finishing their education in Paris or Berlin.

In our issue of last week we commented upon a case of compulsory removal to a fever hospital in Aberdeen, under the impression that it had been finally disposed of by the Courts. We regret to find that this case is still *sub judice*, although the report in the local press seemed to leave no doubt on the subject. Scotch legal procedure has always proved a dark mystery to southerners, and the present case is an illustration of the truth of this fact.

A most successful concert on behalf of the Poplar Hospital for Accidents was arranged recently by the Canning Town Committee in the Royal Albert Music Hall. The hall was crowded and proceedings enthusiastic, and the result, both direct and indirect, could not be otherwise than highly beneficial to the hospital. The Hon. Sydney Holland was present and took an active and appreciated part in the proceedings, both by delivering a speech and showing views illustrative of the hospital and its work.

The Supervising Council of the Paris Assistance Publique has framed a new tariff for paying patients in the hospitals of Paris. The alteration is directed towards making a distinction between charges for medical and surgical treatment. The original sum of 3 francs 30 centimes per diem for either, without distinction, will be required from medical patients, whilst the higher charge of 5 francs will be made for surgical cases. The payments for children have been altered proportionately, and those for lying-in cases have been raised from 3 francs 30 c. to 5 francs.

We congratulate St. George's Hospital upon the election of Mr. Timothy Holmes to the important office of treasurer. Mr. Timothy Holmes has given many of the best years of his life to St. George's Hospital as one of the hon. medical staff; he has never failed to take the most genuine interest in everything tending to promote the advancement of sound hospital administration, and we recognise his appointment as a new departure on the part of the lay governors of hospitals which we hope will be widely followed. It not infrequently happens that when an honorary physician or surgeon retires from office, and is elected to the office of consulting physician or surgeon, he has good health, has done the greater portion of his work in his profession, and so could, and would, we believe, be glad, to give of his time to the management of the hospital where he has spent so many years of his life. Why should not consulting physicians and surgeons be elected to the offices of chairman of committee or treasurer to our great voluntary hospitals, when they show the capacity for such work? In any case, we welcome Mr. Timothy Holmes' appointment most cordially, and congratulate him upon his election to so responsible an office as that of Treasurer to St. George's Hospital.

The Rev. John Henn has retired from the office of secretary to the Manchester Hospital Saturday and Sunday Funds, which he has held for twenty-five years. The Lord Mayor moved a resolution expressing great regret at the loss of Mr. Henn's services, testifying to the earnestness, fidelity, and zeal which he has displayed in the management of the work, by which the sum of £187,676 has been raised for the medical charities of Manchester and Salford. Mr. Henn was elected a member of the committee, and Mr. T. R. Wilkinson and Mr. Popplewell have accepted office as treasurer and secretary to a proposed testimonial fund for the Rev. J. Henn. Mr. Henn has deserved well of Manchester, its city and inhabitants; he has shown a lively interest in the development of the two funds there during the last twenty-five years, and should have every credit for their success. Mr. Henn, at one time, seemed disposed to claim to be the founder of the Hospital Sunday itself, but we are glad to see that in face of the evidence to the contrary he has not proceeded in a claim which was not to his credit, and which has since been put forward on his own behalf by Alderman Holmes of Newcastle-on-Tyne, who states that he first suggested a Hospital Sunday there in 1869. As we have proved more than once in these columns, Hospital Sunday originated in the periodical collections which were established by the late Rev. Canon Miller in Birmingham, in 1858, at the suggestion of the late Mr. T. B. Wright. This was eleven years earlier than Alderman Holmes' first letter on the subject; and four years earlier, namely, on October 28th, 1865, the *Birmingham Daily Post* commenced a leading article by describing the collections at Birmingham on the following Sunday as "Hospital Sunday." It follows that Birmingham is entitled to the honour of having originated and founded Hospital Sunday. How any person now living can venture to claim the honour seeing that Canon Miller and Mr. Wright are both dead, in the face of the documentary evidence published in these columns and elsewhere, we cannot imagine.

